# Low testosterone state inhibits erectile function by downregulating the expression of GIT1 in rat penile corpus cavernosum

**DOI:** 10.1093/sexmed/qfad017

**Published:** 2023-05-29

**Authors:** JianBin Gu, Li-kun Zhu, Xin Zhao, Jun Jiang, Rui Jiang

**Affiliations:** Department of Urology, The Affiliated Hospital of Southwest Medical University, Luzhou, 646000, China; Department of Urology, The Affiliated Hospital of Southwest Medical University, Luzhou, 646000, China; Department of Urology, The Affiliated Hospital of Southwest Medical University, Luzhou, 646000, China; Department of Thyroid Surgery, The Affiliated Hospital of Southwest Medical University, Luzhou, 646000, China; Department of Urology, The Affiliated Hospital of Southwest Medical University, Luzhou, 646000, China; Department of Urology, Nephropathy Clinical Medical Research Center of Sichuan Province, Luzhou, 646000, China

**Keywords:** androgen, hypoandrogenism, G protein–coupled receptor kinase interactor 1 (GIT1), nitric oxide synthase, protein-protein interaction

## Abstract

**Background:**

The mechanism of erectile dysfunction (ED) caused by a low androgen level is still not clear.

**Aim:**

To explore the influence of the low testosterone state on G protein–coupled receptor kinase interactor 1 (GIT1) and its contact to erectile function.

**Methods:**

Thirty male Sprague-Dawley rats aged 8 weeks were distributed at random into 5 groups: control (sham operated), castration, testosterone supplement after castration, castration + vacant lentiviral transfection, and castration + lentiviral transfection. The testis and epididymis were removed through a scrotal incision to develop castrated rats. Four weeks after castration, a lentivirus carrying the *GIT1* gene was injected into the middle of rat penile corpus cavernosum. One week after transfection, maximum intracavernous pressure/mean arterial pressure (ICPmax/MAP), serum testosterone, nitric oxide, GIT1, endothelial nitric oxide synthase (eNOS), phospho-eNOS (p-eNOS), p-eNOS/eNOS, and the interaction between eNOS and GIT1 were assessed in the rats.

**Outcomes:**

The levels of GIT1 in the penile cavernous tissue of castrated rats are significantly lower than that of controls.

**Results:**

GIT1 was expressed in the cytoplasm and cell membrane of vascular endothelial cells and smooth muscle cells in rat penile tissue. In comparison with normal rats, the castrated rats showed lower levels of GIT1 expression, GIT1 and eNOS interaction, p-eNOS/eNOS, nitric oxide, and ICPmax/MAP (*P* < .01). Overexpression of GIT1 can intensively enhance the expression level of GIT1, the interaction between GIT1 and eNOS, p-eNOS/eNOS, nitric oxide, and ICPmax/MAP in rats (*P* < .01).

**Clinical Translation:**

Modulating the interaction between eNOS and GIT1 might be a novel method of treating ED caused by a low androgen level.

**Strengths and Limitations:**

The impact of GIT1 phosphorylation on the activity of eNOS and its possible mechanisms affecting erectile function require further study.

**Conclusion:**

A low testosterone state inhibits erectile function in rats by reducing the expression of GIT1 and the protein interaction between GIT1 and eNOS.

## Introduction

Erectile dysfunction (ED) is defined as insufficient penis erection for satisfactory sexual activity. As a result of the global trend of aging, ED is now an unavoidable issue for elderly men. The prevalence of ED increases considerably as age increases. By the age of 70 years, the incidence rate of ED in the population can reach 40%.^1^ Testosterone has an important influence on regulating the integrity and function of the cavernous tissue of the penis, and the reduced serum testosterone level in men due to a variety of causes is one etiology of ED.^2^ Although patients with ED and low testosterone levels may experience an improvement in erectile function after testosterone supplement therapy, the effectiveness of the treatment is limited. Many patients have to maintain a hypoandrogen state (eg, prostate cancer treatment with androgen deprivation). Therefore, finding a more effective treatment for these patients with ED remains an important research issue.

The nitric oxide (NO) pathway is the main signal transduction pathway during the physiologic process of penile erection. In the corpus cavernosum, endothelial NO synthase (eNOS) catalyzes L-arginine to produce NO, which promotes the conversion of cGMP to relax the smooth muscle cells and blood vessels in corpus cavernosum, thus initiating penile erection.^3^ As an essential component of the eNOS/NO/cGMP pathway, eNOS activity is controlled by many factors, including eNOS coupling, posttranslational modification, and protein-protein interactions.^4^ Protein-protein interactions are an essential mechanism for the posttranslational regulation of eNOS. eNOS can interact with a variety of regulatory and structural proteins, such as Hsp90, CaM,^5^^,^^6^ caveolin 1, and NOSIP,^7^^,^^8^ resulting in the promotion or inhibition of the formation and release of NO. Research has revealed that low testosterone levels may diminish NO production and impair erectile function by eNOS inactivity and endothelial dysfunction. However, the particular mechanism has not been clarified.^9^

G protein–coupled receptor kinase interactor 1 (GIT1) is a GTP-activating protein. GIT1 also acts as a part of the scaffold protein complex to link signaling molecules in cells to different sites of action.^10^^,^^11^ GIT1 is a new mediator that affects thrombin-mediated signal transduction, endothelial cell contraction, and permeability, and it participates in the regulation of endothelial cell barrier function.^12^^,^^13^ At the same time, GIT1 is an important regulator of vascular remodeling, which is essential for the proliferation, migration, and apoptosis of vascular smooth muscle cells.^14^ As a recently discovered factor that interacts with eNOS, GIT1 can interact with eNOS resulting in phosphorylation of eNOS (Ser1177). This can promote NO synthesis.^15^ In a rat model of preeclampsia, GIT1 knockdown can inhibit the activity of eNOS in the placenta and induce the exacerbation of the preeclampsia phenotype.^16^ In a rat model of hepatic sinusoidal vascular endothelial injury, the expression level of GIT1 in vascular endothelial cells is decreased; the interaction between GIT1 and eNOS is weakened; and the activity of eNOS and the expression of NO is decreased. However, upregulation of GIT1 in damaged hepatic sinusoidal vascular endothelial cells can directly activate eNOS and considerably increase NO expression.^17^ How do low testosterone levels affect the expression of GIT1 in the penile corpus cavernosum of rats to inhibit erectile function? At present, the relationship between GIT1 and androgen is still unclear. Therefore, understanding the potential influence of hypoandrogenism on GIT1 expression and the interaction between GIT1 and eNOS in the corpus cavernosum may provide a foundation for finding a novel treatment for ED.

## Methods

### Animals

Thirty male Sprague-Dawley rats (Animal Center; Southwest Medical University) aged 8 weeks were distributed at random into 5 groups: control (sham operated), castration, testosterone supplement after castration, castration + vacant lentiviral transfection, and castration + lentiviral transfection. The testes and epididymis were surgically removed from a castrated rat model. One day after the operation, the rats in the testosterone supplement group received subcutaneous injections of testosterone propionate (3 mg/kg) every 2 days. At the end of the fourth week, the rats were anesthetized for lentiviral transfection. After a rubber band was tied around the base of the penis, castrated rats were injected into the middle of the corpus cavernosum with a lentivirus vector to overexpress GIT1.^18^ A vacant lentiviral vector was used as a control. Lentivirus titers were consistent in all groups (1 × 10^8^ transduction units/mL, 10 μL).

The experimental rats were housed in a standard animal laboratory. The temperature of the environment was 22 to 28 °C, and the relative humidity was 40% to 60%. The rats could drink and eat freely. All experimental procedures were done according to the guidelines of the Chinese Society of Laboratory Animals and the *Guide for the Care and Use of Laboratory Animals* (US National Institutes of Health, publication 85-23, revised 1996).

### Determination of the maximum intracavernous pressure/mean arterial pressure and serum testosterone

Four weeks after castration surgery, the rats were anesthetized. Mean arterial pressure (MAP) and intracavernous pressure (ICP) were monitored by pressure transducers connected to the computer (BL-420S Biological Function Experiment System; Chengdu Instrument Factory). The cavernosal nerve was stimulated with electrodes at different intensities (0, 3, and 5 V).^19^ The changes of maximum ICP (ICPmax)/MAP during stimulation were recorded by a computer biological acquisition system. After that, 2 mL of blood were drawn from the carotid artery to test the concentration of serum testosterone via the Rat Serum Testosterone Elisa Kit (Elabscience).

### Lentivirus transfection efficiency

The cavernous tissue of the rat penis was frozen, fixed, and sectioned. Sections of frozen corpus cavernosum tissue were stained with diamidine phenylindole. The stained slices were observed with a fluorescence microscope. Cells with green fluorescence under blue light excitation demonstrated positive transfection. The number of green cells and blue cells were calculated with Image-Pro Plus 6.0 (Media Cybernetics Inc), and the ratio of green fluorescence to blue fluorescence was determined as lentiviral transfection efficiency.^20^

### Immunohistochemistry staining

In brief, after fixing, embedding, slicing, repairing, and sealing, the primary antibodies were added and incubated with sections of penile cavernous tissue: anti-GIT1 antibody (1:100; Abcam), anti-eNOS antibody (1:200; Abcam), and anti-phospho-eNOS-Ser^1177^ antibody (1:500; Abcam). After overnight incubation at 4 °C, the horseradish peroxidase–conjugated secondary antibody (1:1000; Proteintech) was added.^21^ Coloring agents were added to render different colors. Brown-yellow staining represents positive expression. Specific signals were scanned and quantitated with Image-Pro. The integrated optical density was used to represent protein expression levels.

### Western blotting

To extract the protein, the penile spongy tissue was pulverized and added into the radioimmunoprecipitation assay lysis buffer containing enzyme inhibitors (Beyotime Biotechnology). The bicinchoninic acid protein concentration measurement kit (Beyotime Biotechnology) was used to quantify the protein content of supernatants after centrifugation. The supernatant was mixed with a loading buffer and heated to 100 °C to denature the protein. The samples were stored at −20 °C. After electrophoresis, transmembrane, and membrane closure, the polyvinylidene difluoride membranes were incubated with primary antibodies at 4 °C overnight: anti-eNOS antibody (1:1000; Abcam), anti-phospho-eNOS-Ser^1177^ antibody (1:1000; Abcam), and anti-GIT1 antibody (1:100; Santa Cruz Biotechnology). After the membranes were rinsed, the horseradish peroxidase–conjugated secondary antibody (1:1000; Proteintech) was added and incubated at room temperature for 1 hour. The protein strips were presented by the protein imager (Bio-Rad Laboratories) after addition of the enhanced chemiluminescence solution. The grayscale value of the protein strips was quantitated with QuantityOne 4.6 software (Bio-Rad Laboratories).^19^^,^^22^

### Coimmunoprecipitation

Fresh penile tissue was pulverized and added into the immunoprecipitation lysis solution containing specific enzyme inhibitors (Beyotime Biotechnology) to extract the protein. The bicinchoninic acid protein concentration measurement kit (Beyotime Biotechnology) was used to quantify the protein content of supernatants after centrifugation. The supernatant was incubated for 1 hour at 4 °C with rabbit common IgG (Santa Cruz) and protein A + G agarose (Santa Cruz). After centrifugation, anti-eNOS antibody (Abcam) and resuspended agarose (Santa Cruz) were added to the supernatant, and the whole thing was incubated at 4 °C overnight. Then, the sample was centrifuged for 5 minutes to collect the immunoprecipitates. The immunoprecipitates were washed 5 times with the immunoprecipitation lysis solution, each time repeating the centrifugation step. After the final wash, the supernatant was removed; loading buffer was added; and the protein was denatured by boiling and stored at −20 °C. The remaining procedures were the same as those used for Western blotting.

### NO content determination

NO concentration was measured with the NO biochemical test kit (Elabscience) according to the instructions of the manufacturer: fresh penile tissue was pulverized, homogenized with 2 to 8 °C phosphate-buffered saline (0.01 M, pH 7.4), and centrifuged at 4 °C (10 000 × *g* for 10 minutes). The supernatant and standard dilutions were added to the corresponding well. The optical density of each well was evaluated with a microplate reader (TECAN Infinite M200). The NO concentration was calculated with the data-established standard curve.

### Statistical methods

All data were statistically analyzed by Prism Software version 8.2.1 (GraphPad Software) and expressed as mean ± standard deviation. Differences between samples were processed by 1-way analysis of variance and linear correlation analysis. *P* < .05 was regarded as statistically significant.

## Results

### Body weight, serum testosterone, and ICPmax/MAP

All groups of rats had similar weights. Castration decreased the serum testosterone levels of rats (1.52 ± 0.18 nmol/L) vs the sham-operated group (21.92 ± 1.71 nmol/L) and the testosterone supplement group (22.61 ± 1.96 nmol/L). However, the serum testosterone level of rats did not substantially change after transfection of the *GIT1* gene (1.44 ± 0.16 nmol/L) vs the castrated rats (1.52 ± 0.18 nmol/L). The erectile function of rats was evaluated by ICPmax/MAP. The ICPmax/MAP was considerably decreased after castration (0.34 ± 0.02, 3 V; 0.40 ± 0.01, 5 V) and recovered after androgen supplementation (0.57 ± 0.01, 3 V; 0.65 ± 0.03, 5 V) vs the sham-operated group (0.56 ± 0.03, 3 V; 0.67 ± 0.03, 5 V). Yet, ICPmax/MAP was obviously increased after overexpression of GIT1 (0.43 ± 0.02, 3 V; 0.53 ± 0.03, 5 V) vs the castrated group (0.34 ± 0.02, 3 V; 0.40 ± 0.01, 5 V) (*P* < .01; Figure S1).

**Figure 1:**
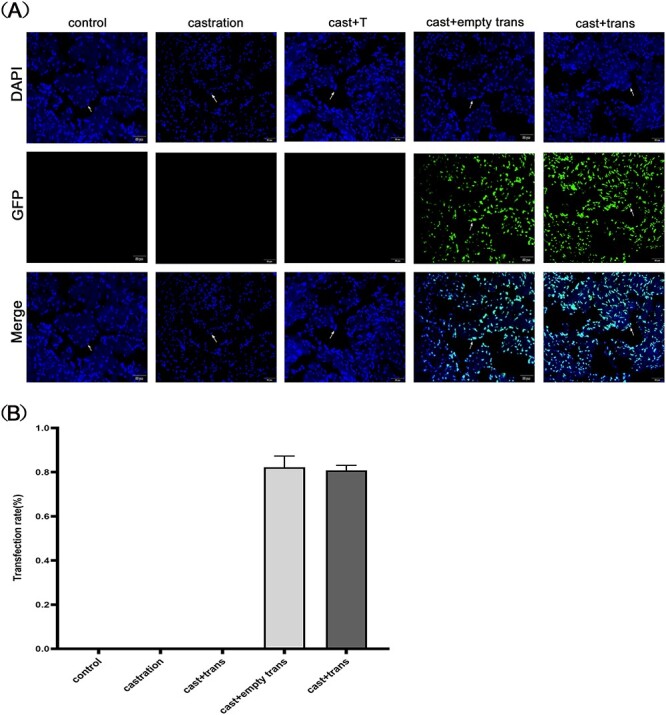
The expression of GIT1 in the penile cavernosum of transfected rats by immunofluorescence. (A) The successfully transfected cells fluoresced green under blue light stimulation and were mostly found in the endothelial cells of corpus cavernosum (white arrows). (B) The efficiency of lentivirus transfection in rat corpus cavernosum. GIT1, G protein–coupled receptor kinase interactor 1.

### Fluorescence staining

To investigate the impact of GIT1 on eNOS activity, we overexpressed the *GIT1* gene in the corpus cavernosum of castrated rats with a constructed lentiviral vector containing specific sequences. The successfully transfected cells fluoresced green under blue light stimulation and were mostly found in the endothelial cells of the corpus cavernosum. Groups without transfection showed no green fluorescence (Figure 1).

### Immunohistochemistry staining

**Figure 2:**
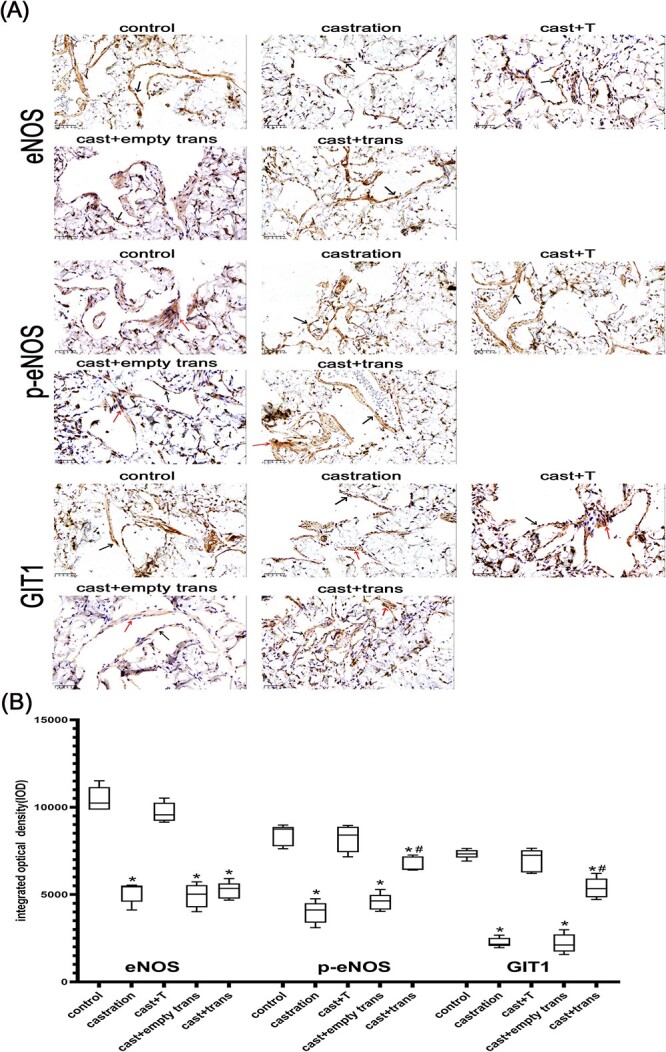
Immunohistochemical localization of GIT1, eNOS, and p-eNOS in the corpus cavernosum of rats. (A) Positive expression was immunolabeled as brown-yellow staining. GIT1 was mainly expressed in the cytoplasm of vascular endothelial cells (black arrows) and smooth muscle cells (red arrows). (B) Integrated optical density was used to represent protein expression levels. ^*^P < .01 vs sham-operated and testosterone supplement groups. ^#^*P* < .01 vs castration and vacant lentiviral transfection groups. eNOS, endothelial nitric oxide synthase; GIT1, G protein–coupled receptor kinase interactor 1; p-eNOS, phospho-eNOS.

GIT1, eNOS, and p-eNOS were mainly expressed in the cytoplasm and cell membrane of vascular endothelial cells and smooth muscle cells in rat penile tissue (Figure 2). The expression levels of GIT1, eNOS, and p-eNOS were considerably decreased after castration (*P* < .01). In contrast, GIT1 and p-eNOS expression levels were increased after transfection of the *GIT1* gene (*P* < .01). However, when compared with that of castrated rats, the expression of eNOS did not change considerably in the castration + lentiviral transfection group.

### Measurement of protein concentrations by Western blot

**Figure 3:**
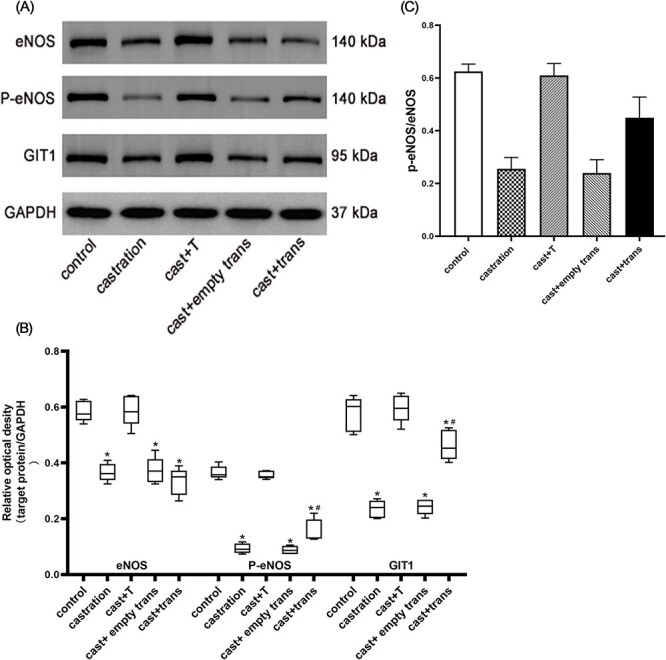
The expression levels of GIT1, eNOS, and p-eNOS in the corpus cavernosum of rats. (A) Representative images of protein expression by Western blotting. (B) Relative optical density was applied to determine the level of protein expression. (C) The activity of eNOS is presented by p-eNOS/eNOS. The expression levels of GIT1, eNOS, p-eNOS, and p-eNOS/eNOS in castrated rats were decreased when compared with those of the sham-operated and testosterone supplement groups (^*^P < .01). The expression of GIT1, p-eNOS, and p-eNOS/eNOS increased considerably after overexpression of the GIT1 gene when compared with that of the castration and no-load lentiviral transfection groups (^#^*P* < .01). eNOS, endothelial nitric oxide synthase; GIT1, G protein–coupled receptor kinase interactor 1; p-eNOS, phospho-eNOS.

The expression levels of GIT1, eNOS, and p-eNOS were considerably decreased after castration (*P* < .01). After introduction of the lentivirus carrying the *GIT1* gene, the expression of GIT1 and p-eNOS and the ratio of p-eNOS/eNOS increased considerably (*P* < .01). However, there was no significant difference in eNOS expression level between the castration group and the castration + lentiviral transfection group (Figure 3).

### Protein interaction between GIT1 and eNOS

**Figure 4:**
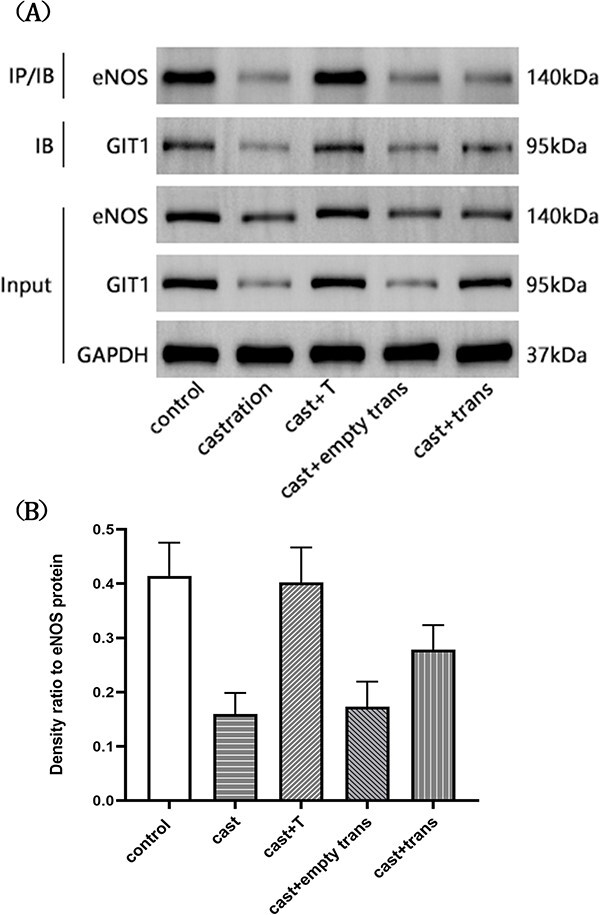
The protein interaction between GIT1 and eNOS in the corpus cavernosum of rats. (A) Representative images of protein-protein interaction by immunoprecipitates (IP). (B) Densitometric ratio of GIT1 to eNOS protein. The interaction between GIT1 and eNOS was considerably weakened after castration when compared with that of the sham-operated and testosterone supplement groups (^*^P < .01). The interaction between GIT1 and eNOS was obviously enhanced after overexpression of the GIT1 gene when compared with that of the castration and no-load lentiviral transfection groups (^#^*P* < .01). eNOS, endothelial nitric oxide synthase; GIT1, G protein–coupled receptor kinase interactor 1.

The interaction between GIT1 and eNOS was considerably weakened after castration (*P* < .01). It may have been caused by decreased expression of GIT1 or a low testosterone state. After introduction of the lentivirus carrying the *GIT1* gene, the interaction between GIT1 and eNOS was obviously enhanced (*P* < .01; Figure 4).

### Concentration of NO

NO levels in rats were obviously reduced after castration (9.89 ± 0.93 μmol/g, *P* < .01). After transfection of the GIT1 gene, the level of NO (14.87 ± 1.69 μmol/g) in the corpus cavernosum was obviously higher than that of the castrated rats (9.89 ± 0.93 μmol/g, *P* < .01).

### Correlation analysis

Serum testosterone concentration was positively correlated with GIT1 expression (*Y* = 0.2941 + 0.013*X*, *r* = 0.8258, *P* < .01). The GIT1-eNOS interaction was positively correlated with p-eNOS/eNOS (*Y* = 0.09211 + 1.22*X*, *r* = 0.7632, *P* < .01).

## Discussion

The expression levels of NO and ICPmax/MAP in castrated rats were lower than those of rats in the sham-operated group and the testosterone supplement group. This result suggested that the decreased level of androgen may inhibit the function of erection in rats by reducing the expression of NO. Additionally, testosterone supplementation improved the erectile function of rats. The outcomes are consistent with the experimental results of the previous research group.^23^

This is the first confirmation of the existence of GIT1 in the cytoplasm and plasma membrane of vascular endothelial cells and smooth muscle cells in the penile corpus cavernosum of rats. The expression of GIT1, eNOS, p-eNOS, and p-eNOS/eNOS was considerably lower in castrated rats than in sham-operated rats (*P* < .01). These results indicated that a low testosterone state leads to a decrease in the expression of GIT1, eNOS, p-eNOS, and p-eNOS/eNOS in the corpus cavernosum. However, the expression of GIT1, eNOS, p-eNOS, and p-eNOS/eNOS showed no difference between the sham-operated group and the castration + testosterone group, indicating that testosterone supplementation can inhibit the decrease in GIT1 and eNOS expression and restore eNOS activity. Transfection with the *GIT1* gene considerably increased the expression of GIT1, p-eNOS, and p-eNOS/eNOS as compared with that of castrated rats. GIT1 expression was positively related to p-eNOS/eNOS, NO, and ICPmax/MAP, implying that overexpression of GIT1 in castrated rats may increase eNOS activity, p-eNOS/eNOS, and erectile function. This is consistent with the findings that the upregulation of GIT1 in damaged hepatic sinusoidal vascular endothelial cells considerably increased the activity of eNOS.^17^ Yet, more research is required to explain the biological effects of GIT1 overexpression in a low testosterone state and its relationship with the eNOS/NO pathway. After transfection of the *GIT1* gene in castrated rats, the expression of eNOS showed no significant changes. The findings suggest that GIT1 may not directly affect the expression of eNOS. Instead, the eNOS activity may be regulated by posttranslational modifications, such as phosphorylation, acetylation, S-nitrosation, and protein-protein interactions, thus affecting the NO level in rat penis cavernous tissue and changing its erectile function. Meanwhile, in the study, the ratio of ICPmax/MAP in the GIT1 overexpression group was significantly increased as compared with that in the castrated group but still significantly below the sham group (*P* < .05). This may be related to the regulation of multiple signal transduction pathways during penile erection.

In this experiment, the coimmunoprecipitation results revealed that the protein interaction of GIT1-eNOS was considerably decreased in the castrated rat model, while the GIT1-eNOS interaction was considerably increased after transfection with the *GIT1* gene. Beyond that, eNOS activity, p-eNOS/eNOS ratio, and NO expression were obviously increased after transfection with the *GIT1* gene. This demonstrates that GIT1 may regulate the interaction between GIT1 and eNOS to enhance the activity of eNOS, increase the p-eNOS/eNOS ratio, and ultimately increase the production of NO. This is consistent with the findings that the upregulation of GIT1 in the damaged hepatic sinusoidal vascular endothelial cells considerably increased the GIT1-eNOS interaction.^17^

Upregulation of GIT1 expression in the penile cavernous tissue of the castrated rat by gene transfection can improve the ICPmax/MAP of the rat. However, the ICPmax/MAP in the transfected group was still lower than that in the sham group. This may be due to low androgen status inhibiting erectile function in rats through multiple signal pathways (signal networks), including the GIT1 signal pathway.

Hypoandrogenism decreases the expression of GIT1 and impairs the interaction between GIT1 and eNOS, resulting in a reduction in p-eNOS/eNOS. This suggests that vascular endothelial dysfunction mediated by GIT1 may be one of the mechanisms contributing to ED in hypoandrogenism. The overexpression of GIT1 by transfection improves the GIT1-eNOS protein interaction, increases the activity of eNOS, and ultimately improves erectile function in rats. This study suggests that modulating the interaction between eNOS and GIT1 might provide a novel method of treating ED in the future. Protein-protein interactions are a critical mechanism for the posttranslational regulation of eNOS. Nevertheless, the role of other posttranslational modifications (acetylation, nitrosation, etc) in eNOS activity remains unidentified. Studies have found that GIT1 may also be stimulated by Akt to undergo tyrosine phosphorylation, which can improve the protein interaction of GIT1-eNOS, increase the expression of NO, and improve vascular endothelial function.^24^ Therefore, the impact of GIT1 phosphorylation on the activity of eNOS and its possible mechanisms affecting erectile function are worthy of further study.

## Author contributions

J.G., L.Z., J.J., and R.J. participated in the design of the trial and conducted the data acquisition. J.G., L.Z., J.J., and R.J. drafted and revised the manuscript. J.G., L.Z., X.Z., and R.J. interpreted and analyzed the data. J.G., L.Z., and X.Z. guided the experiment directions and revised the manuscript. J.G. performed the statistical analysis. All authors read and approved the final version of the manuscript.

## Funding

This study was supported by the Research Foundation of the Department of Human Resources and Social Security of Sichuan Province of Returned Scholars (grant 2019-76).

*Conflicts of interest:* None.

## Supplementary Material

Figure_1_supplement_qfad017
