# Supplementary material for: Low testosterone state inhibits erectile function by downregulating the expression of GIT1 in rat penile corpus cavernosum
Source: Sex Med. 2023 May 29;11(2):qfad017. doi: 10.1093/sexmed/qfad017 (PMC10226814; doi:10.1093/sexmed/qfad017)
Supplement: Figure_1_supplement_qfad017 [file figure_1_supplement_qfad017.docx]

Supplement material:


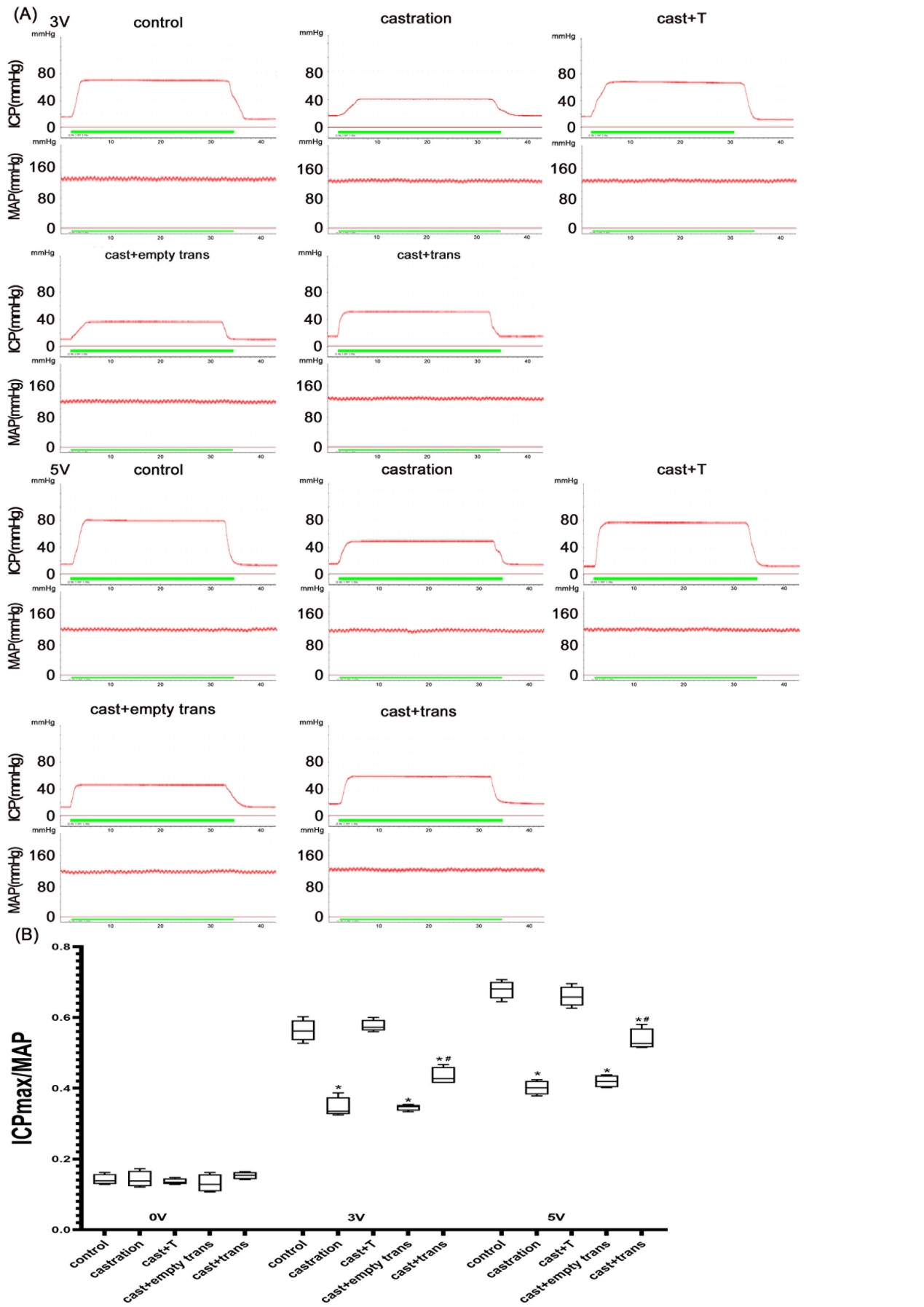


**Figure 1** **Supplement** Eretile response of rats was measured by different intensity of electrostimulation. Panel (A) depicts [the variation tendency](https://dict.youdao.com/w/eng/the_variation_tendency/#keyfrom=dict.phrase.wordgroup) of ICP and MAP at 3 V and 5 V electrostimulation by curve graph. Panel (B) indicates the ratio of maximum ICP (ICPmax) to MAP in rats of each group. *p<0.01 and #p<0.01.
